# Alarming patterns of moderate and high-risk alcohol use among transgender women in Goiás, Central Brazil

**DOI:** 10.3389/fpubh.2024.1333767

**Published:** 2024-02-14

**Authors:** Larissa Silva Magalhães, Kamila Cardoso dos Santos, Bruno Vinícius Diniz e Silva, Gabriel Francisco Silva Filho, Krishna Vaddiparti, Roxana Isabel Cardozo Gonzalez, Sandra Cristina Pillon, Megmar Aparecida dos Santos Carneiro, Karlla Antonieta Amorim Caetano, Robert Lewis Cook, Sheila Araújo Teles

**Affiliations:** ^1^Faculty of Nursing, Federal University of Goiás, Goiânia, Goiás, Brazil; ^2^Institute of Tropical Pathology and Public Health, Federal University of Goiás, Goiânia, Goiás, Brazil; ^3^Department of Epidemiology, University of Florida, Gainesville, FL, United States; ^4^Faculty of Nursing of Ribeirão Preto, University of São Paulo, São Paulo, Brazil

**Keywords:** at-risk population, high-risk alcohol use, alcohol consumption, substance use, transgender women

## Abstract

**Background:**

Scant studies have examined alcohol consumption among transgender women in Latin America. This cross-sectional study estimated the prevalence and associated factors of risky alcohol use among transgender women in Goiás, a state located in the center of Brazil.

**Methods:**

Participants were 440 transgender women (median age = 35 years, interquartile range = 9) recruited through respondent-driven sampling. All participants were interviewed about sociodemographic characteristics, violence, and risk behavior. Alcohol use was assessed using the alcohol use disorders identification test (AUDIT). An AUDIT score greater than or equal to eight was considered as risky alcohol consumption. Logistic regression analysis was used to examine predictors of risky alcohol use, and *p*-values <0.05 were considered significant.

**Results:**

The majority were young, single, sex workers. Most transgender women had used alcohol in the previous year (85.7%), and more than half (56.6%) reported binge drinking and risky alcohol consumption (60.2%). There was a high overlap between sexual behavior, drugs, and alcohol use. Using alcohol during sex (adjusted odds ratio [aOR]: 2.9; 95% confidence interval [CI]: 1.7–4.8), cocaine/crack use (aOR: 2.3; 95% CI: 1.5–3.7) and having a drug user as a sexual partner (aOR: 2.9; 95% CI: 1.5–5.9) were independently associated with risky alcohol consumption.

**Conclusion:**

Alcohol consumption was highly prevalent, and drugs seem to play an important role in risky alcohol consumption among transgender women Goiás. These findings support stakeholders to promote intervention strategies to reduce this pattern of alcohol consumption and reduce the burden of substance use disorders among transgender women.

## 1 Introduction

The global burden of disease (GBD) study estimates that 59.1% of people drink unsafe amounts of alcohol and 1.34 billion people have a harmful pattern of alcohol use (1). In addition, more than 35 million people experience health-related problems owing to the use and misuse of alcohol. Alcohol use accounts for 5.1% of the GBD and high rates of alcohol-related hospitalization and mortality from acute and chronic non-communicable diseases. Alcohol is also the leading risk factor for death in the population aged 15–49 years (1).

Research has found a high prevalence of alcohol use among lesbian, gay, bisexual, and transgender (LGBT) populations (2, 3). However, this is not homogenous in the diverse LGBT individuals. Some authors have shown higher rates of alcohol use among transgender women compared to other sexual minorities (4, 5). In California, United States, a study of young middle- and high-school students found that the prevalence of substance use among transgender students was 2.5- to 4-times higher than among non-transgender students (6).

From a very young age, transgender women experience violence, prejudice, and stigma daily (7, 8). These negative experiences are associated with high-risk alcohol use. Alcohol use can impair safe sex decision-making (9) and have been associated with sexually risky behaviors such as unprotected sex and multiple sex partners (10). These events may contribute to the great prevalence of human immunodeficiency virus (HIV) and other sexually transmitted infections (STIs) among transgender women (11).

Studies have shown a high prevalence of high-risk alcohol use among transgender women, ranging from 9.2 to 48.2% (2, 4, 12). However, most of the research on alcohol use among transgender women has been conducted in developed countries, particularly the United States (13). In Brazil, a Latin American country with the highest number of transgender homicides (14), there is only one study of alcohol use among this key population, in which 48.2% of transgender women (*n* = 304) showed a pattern of risky alcohol use (2). However, given the vast size of the country and its socioeconomic and cultural diversity, this prevalence could not be representative of the country as a whole.

According to Gilbert et al. (4), effective risk reduction efforts among transgender women still depend on scientific efforts to identify sex and gender associations with alcohol use to develop effective strategies to reduce STIs and alcohol-related problems. Despite the recent increase in studies involving sexual minorities, there are still gaps in the literature regarding patterns in the use of alcohol and other substances.

A widely used method to identify patterns of alcohol use is the alcohol use disorders identification test (AUDIT), which has been validated in several countries globally and is easy to administer (15). The present study estimates the prevalence and determines the association of sociodemographic characteristics, sexual behavior, and other substance use with risky alcohol use among transgender women in Goiás, Central Brazil. These findings add information concerning alcohol consumption in a key population in developing countries, contributing to the formulation of policies, programs, prevention strategies, and early interventions related to the management of substance abuse.

## 2 Methods

### 2.1 Study design

This cross-sectional study included 440 transgender women in three cities in Goiás, a mid-western region of Brazil. It was approved by the research ethics committee of the Universidade Federal de Goiás-UFG (no. 2.358.818).

### 2.2 Population and location

An estimated 0.69% of adults in Brazil are transgender people (16). The study population consisted of transgender women who either lived or were in transit in the three cities in Goiás: Metropolitan region of Goiânia (2,173,006 inhabitants; Human Development Index [HDI]: 0.862), Itumbiara (104,742 inhabitants; HDI: 0.752), and Jataí (100,882 inhabitants; HDI: 0.757). These cities are an important economic hub and are also the routes for prostitution and sex tourism in the region (17, 18).

Transgender is an umbrella term used to define persons whose gender assigned at birth differs from their identity, expression, or behavior (19). The parameters considered for the sample size calculation of 384 participants were necessary to exhibit statistical significance, considering the prevalence of risky alcohol consumption of 48% (7), a significance level of 95%, and a precision of 5.0%. Eligible participants were individuals who defined themselves as transgender women to the study recruiter at the time of enrollment and presented a valid respondent-driven sampling (RDS) invitation. Those under the influence of psychoactive drugs at the time of the interview were excluded.

### 2.3 Data collection

Transgender women are considered a hard-to-reach population (20); therefore, the RDS method was used to recruit participants. This method is a variant of the snowball sampling method in which participants are recruited by peers. It is widely used in studies of key populations (20–22).

As previously described by Ferri et al. (23), all participants were informed about the study objectives, and they provided written informed consent. Data collection started in April 2018 and ended in November 2019. For data collection, face-to-face interviews were conducted by trained members of our research team. For this purpose, it was used a standardized structured questionnaire based on “diversity and valorization of life” research (20), and to assess the consumption of alcohol and other drugs (AUDIT and alcohol, smoking, and substance involvement screening test).

### 2.4 Variables

For the selection of predictive variables, the context of vulnerability of transgender women was considered based on publications involving substance use in sexual minorities (2, 3).

#### 2.4.1 Sociodemographic data

We divided the data by age group (16–20; 21–24; 25–30 or > 30 years), marital status single (yes or no), race/ethnicity (White, mixed-race, or Black), and years of education (< 9, 10–12, or ≥ 13). Education was categorized by the education grades in Brazil (fundamental grade is equivalent to 9 years of school, secondary grade = 12 years, and undergraduate = at least 16 years), and monthly income in US dollars (USD) (≤255, 255–767, or ≥ 767). During the study period, the monthly minimum wage was equivalent to approximately 255 USD.

#### 2.4.2 Gender-related experiences

We collected data on gender-related experiences. When asked about their current job, participants who responded “prostitute,” “sex worker,” “commercial sex worker,” or “call girl” were classified as a “sex worker.” We also asked, “Have you ever felt excluded from your family because of your gender identity?” Participants responded “yes” or “no.” Lastly, we asked, “Have you ever been a victim of physical violence?” and “Have you ever been a victim of sexual violence?” Participants responded “yes” or “no.”

#### 2.4.3 Sexual behavior

Data were also collected on the number of sexual partners during the past 7 days, according to category (none or 1 partner, 2–10 partners, or > 10 partners); sex under the influence of alcohol (“Have you ever had sex under the influence of alcohol or drugs?” [yes or no]); and sex with drug-using partners (“Have you ever had sexual intercourse with a partner who uses drugs?” [yes or no]).

#### 2.4.4 Substance use

Lifetime use of tobacco, cocaine/crack, and marijuana was assessed by asking the questions: “In your life, have you ever used tobacco?” [yes or no]; “In your life, have you ever used cocaine/crack?” [yes or no]; and “In your life, have you ever used marijuana?” [yes or no].

##### 2.4.4.1 Patterns of alcohol use

The AUDIT comprises 10 questions to assess alcohol consumption during the preceding year. The score ranges from 0 to 40 and consumption patterns are divided into two categories: low alcohol risk/no use = 0–7, and risky alcohol consumption = ≥ 8 (15). This study used a dichotomous outcome of an AUDIT score equal to or greater than eight to indicate risky alcohol consumption. Binge drinking was defined as the ingestion of five or more alcoholic beverages on one occasion (24).

### 2.5 Data analysis

Data were collected and entered EpiData version 3.1 (“The EpiData Association” Odense, Denmark) and exported to SPSS 28.0 (IBM, SPSS Statistics; Armonk, NY, United States). We used RDS sampling, but we did not use RDS inference for data analysis because the performance of this method remains unknown (25). Prevalence with a 95% confidence interval (CI) was estimated for all the categorical variables. A chi-squared test was used to identify associations between predictor factors and risky alcohol consumption.

Forward logistic regression model was used to assess the adjusted associations of variables with *p* < 0.20 in the bivariate analysis, and variables in the final model with a *p*-value of <0.05 were considered significant. The fit of the logistic regression models was verified by using the Hosmer and Lemeshow method (*p* = 0.988) (26).

## 3 Results

A total of 440 transgender women participated in this study: 285 in Goiânia, 74 in Itumbiara, and 81 in Jataí. Table 1 shows the characteristics and alcohol use pattern of the study participants. Most participants were younger than 30 years (76.4%), mixed race or black (71.2%), had 10–12 years of schooling (61.4%), and were single. Only 23.6% of participants reported a monthly income of more than 767 USD. Of the participants, 58.6% were sex workers; 53.7% reported that they had been rejected by their family; 40.4% had experienced physical violence; and 48.2% had experienced sexual violence. Study participants reported a high number of sexual partners, with 43.3% reporting more than 10 partners in the past 7 days. History of having a drug-using sexual partner, unprotected anal sex, and sex under influence of alcohol were reported by 87.1, 63.8, and 75.5% of participants, respectively. The majority had used tobacco (59.8%), marijuana (68.9%), and cocaine/crack (59.8%).

**Table 1:** Characteristics and alcohol use pattern among transgender women (*n* = 440) in Goiás, Central Brazil, 2018–2019.

Variable	*n*	%	95% CI*
*Age group*			
16–20	81	18.4	15.1–22.3
21–24	125	28.4	24.4–32.8
25–30	130	29.5	25.5–34.0
>30	104	23.6	19.8–27.8
*Race^a^*			
White	90	20.5	17.0–24.6
Mixed race or black	312	71.2	66.8–75.3
Indigenous or Asian	36	8.2	6.0–11.2
*Schooling (years)*			
≤ 9	48	10.9	8.3–14.2
10–12	270	61.4	56.7–65.8
≥13	122	27.7	23.8–32.1
*Monthly income (US dollars)^b^*			
≥767	104	23.6	19.9–27.8
255–767	211	48.0	43.3–52.6
≤ 255	125	28.4	24.4–32.8
*Single^a^*	376	85.6	82.1–88.6
*Sex work*	258	58.6	54.0–63.1
*Ever experienced physical violence^a,c^*	177	40.4	35.9–45.1
*Ever experienced sexual violence^a,c^*	211	48.2	43.5–52.9
*Ever experienced family rejection^a^*	234	53.7	49.0–58.3
*Number of sexual partners in the last seven days*			
None or 1	146	33.2	28.9–37.7
2–10	103	23.4	19.7–27.6
> 10	191	43.4	38.9–48.1
*Unprotected anal sex^a^*	275	63.8	59.2–68.2
*Sex under influence of alcohol^a^*	330	75.5	71.3–79.3
*Use of cocaine/crack^c^*	263	59.8	55.1–64.3
*Use of marijuana^c^*	303	68.9	64.4–73.0
*Tobacco^c^*	316	71.8	67.4–75.8
*Drug user sexual partner^a,c^*	377	87.1	83.6–89.9
*Alcohol use in the last year*	377	85.7	82.1–88.7
*Binge drinking^d^*	249	56.6	51.9–61.1
*Alcohol use disorders identification test score*			
Low-risk consumption/no use (0–7)	174	39.5	35.1–44.2
Risky consumption (≥ 8)	266	60.5	55.8–64.9

^*^Confidence Interval 95%; ^a^ Variable contain missing data; ^b^ US$ = BRL 3.91 (Dec 2020); ^c^ Lifetime; ^d^ Defined as the ingestion of five or more alcoholic beverages on one occasion for a man or four for a woman.

Considering the alcohol use pattern, 85.7% reported alcohol use in the last year and 56.6% binge drinking. Low-risk consumption or no use was found in 39.8% of the participants and risky consumption in 60.2%.

The prevalence of risky alcohol consumption was significantly higher in participants younger than 25 years, sex workers, and those who reported physical violence (*p* < 0.05). Further, participants with risky alcohol consumption were significantly more likely to report having more than 10 sexual partners in the past 7 days; sex under the influence of alcohol; use of cocaine, marijuana, and tobacco; and having a partner who used drugs (*p* < 0.001; Table 2).

**Table 2:** Bivariate analysis of characteristics associated with risky alcohol consumption in 440 transgender women in Goiás, Central Brazil, 2018–2019.

Variables	Total *n*	Low alcohol risk/no use *n* (%)	Risky alcohol consumption *n* (%)	*p^a^*	OR (95% CI)
*Age (years)*					
16–24	206	68 (33.0)	138 (67.0)		1.0
≥ 25	234	106 (45.3)	128 (54.7)	0.009	0.6 (0.4–0.9)
*Color^b^*					
White	90	37 (41.1)	53 (58.9)		1.0
Mixed race or black	312	123 (39.4)	189 (60.6)	0.772	1.1 (0.7–1.7)
Indigenous or Asian	36	14 (38.9)	22 (61.1)	0.826	1.1 (0.5–2.4)
*Education (years)*					
≤ 9	122	44 (36.1)	78 (63.9)		1.0
10–12	270	108 (40.0)	162 (60.0)	0.463	0.8 (0.5–1.3)
≥ 13	48	22 (45.8)	26 (54.2)	0.247	0.7 (0.3–1.3)
*Monthly income (US$)^c^*					
≥767	175	63 (36.0)	112 (64.0)		1.0
255–767	140	60 (42.9)	80 (57.1)	0.218	0.8 (0.5–1.2)
≤ 255	125	51 (40.8)	74 (59.2)	0.402	0.8 (0.5–1.3)
*Sex work*					
No	182	91 (50.0)	91 (50.0)		1.0
Yes	258	83 (32.2)	175 (67.8)	<0.001	2.1 (1.4–3.1)
*Single*					
No	64	30 (46.9)	34 (53.1)		1.0
Yes	375	144 (38.4)	231 (61.6)	0.200	1.4 (0.8–2.4)
*Ever experienced family rejection^b^*					
No	202	72 (35.6)	130 (64.4)		1.0
Yes	234	101 (43.2)	133 (56.8)	0.110	0.7 (0.5–1.1)
*Ever experienced physical violence^b,d^*					
No	261	113 (43.3)	148 (56.7)		1.0
Yes	177	60 (33.9)	117 (66.1)	0.048	1.5 (1.0–2.1)
*Ever experienced sexual violence^b,d^*					
No	227	94 (41.4)	133 (58.6)		1.0
Yes	211	79 (37.4)	132 (62.6)	0.396	1.2 (0.8–1.7)
*Number of sexual partners in the last seven days*					
≤ 1	146	74 (50.7)	72 (49.3)		1.0
2–10	103	38 (36.9)	65 (63.1)	0.003	1.8 (1.1–2.9)
> 10	191	62 (32.5)	129 (67.5)	<0.001	2.1 (1.4–3.3)
*Unprotected anal sex^b^*					
No	156	62 (39.7)	94 (60.3)		1.0
Yes	275	108 (39.3)	167 (60.7)	0.923	1.0 (0.7–1.5)
*Sex under influence of alcohol^b^*					
No	107	73 (68.2)	34 (31.8)		1.0
Yes	330	99 (30.0)	231 (70.0)	< 0.001	5.0 (3.1–8.0)
*Drug user sexual partner^d^*					
No	56	42 (75.0)	14 (25.0)		1.0
Yes	377	128 (34.0)	249 (66.0)	< 0.001	5.8 (3.1–11.1)
*Use of marijuana^d^*					
No	137	74 (54.0)	63 (46.0)		1.0
Yes	303	100 (33.0)	203 (67.0)	< 0.001	2.4 (1.6–3.6)
*Use of cocaine/crack^d^*					
No	177	103 (58.2)	74 (41.8)		1.0
Yes	263	71 (27.0)	192 (73.0)	< 0.001	3.7 (2.5–5.6)
*Tobacco^d^*					
No	124	66 (53.2)	58 (46.8)		1.0
Yes	316	108 (34.2)	208 (65.8)	< 0.001	2.2 (1.4–3.3)

^a^ Chi-square; ^b^ variable contain missing data; ^c^ US$ 1.00 = BRL 3.91 (Dec 2020); ^d^ lifetime.

These variables and other variables with a *p*-value <0.20 were included in a multivariable logistic regression model. Other variables like having sex under the influence of alcohol (adjusted odds ratio [aOR]: 2.9; 95% CI: 1.7–4.8), using cocaine/crack (aOR: 2.3; 95% CI: 1.5–3.7), and having a drug-user partner (aOR: 2.9; 95% CI:1.5–5.9) remained independently associated with risky alcohol use (Table 3).

**Table 3:** Variables associated with risky alcohol consumption in transgender women in Goiás–GO, Brazil, 2018–2019.

Variable	AOR (95% CI)^a^	*p* value
Sex under influence of alcohol	2.9 (1.7–4.8)	<0.001
Drug-user partner	2.9 (1.5–5.9)	0.003
Cocaine/crack use (lifetime)	2.3 (1.5–3.7)	<0.001

a Adjusted Odds ratio 95% Confidence Interval.

## 4 Discussion

This study estimated the prevalence and factors associated with risky alcohol consumption among transgender women. It is the first study on alcohol use among transgender women in the mid-western region of Brazil. Our findings showed that alcohol use, binge drinking, and risky alcohol consumption were prevalent in this population. Sexual behaviors and cocaine/crack use were associated with risky alcohol consumption.

Almost the totality of transgender women used alcohol in the preceding year, which is much higher as compared to Brazil’s general population (26). In addition, almost two-thirds of the transgender women participating in this study showed risky alcohol consumption. Furthermore, binge drinking, an alcohol consumption pattern associated with unfavorable outcomes such as intoxication, unprotected anal sex, and exposure to violence (5, 27) was found in 56.6% of the transgender women studied. Kerr-Correa et al. (2) also observed a high frequency of risky alcohol consumption among transgender women in Northeast Brazil. Risky alcohol consumption can contribute to the development of alcohol use disorder (AUD) (13, 28, 29). A study conducted in Peru, a neighboring country, reported a prevalence of AUD of 53% among 89 transgender women (29).

In a study conducted in the United States, Staples et al. (30) reported 18% of 317 transgender individuals showed scores of AUDIT indicating risky alcohol consumption, and Gonzalez et al. (31) estimated a prevalence of 21.5% in 680 transgender women. Comparing these data and those reported by Kerr-Correa et al. (2) with our study results, suggests that AUD is more prevalent among transgender women in Brazil than in those in the United States.

Transgender women are subjected daily to several constraints, such as stigma, transphobia, and violence, and these stressors could contribute to risky alcohol consumption (13). In line with this, countries that have policies or laws to protect minorities against hate crimes attributed to homophobia and discrimination tend to have lower rates of alcohol and drug use, risky alcohol consumption, and mental disorders (31). Unfortunately, this is not the case in Latin America and, in particular, Brazil, which ranks first in the ranking of deaths of trans people in the world, which is very likely contributed by the alarming frequency of alcohol consumption in the region (14).

Similar to other studies (13, 32, 33), our findings showed a direct association between cocaine/crack use and risky alcohol consumption. Alcohol causes depression of the central nervous system, including the slowing of cognitive processes (memory, attention, and reaction time) and motor skills, while cocaine increases dopamine levels and the likelihood of impulsive behavior (34).

The interaction between these psychoactive substances produces an active metabolite called cocaethylene, which prolongs the effect of alcohol and cocaine, increases the state of euphoria, and suppresses the sedative effects of alcohol (32, 35). The neurocognitive consequences include executive dysfunction, which is associated with impaired problem-solving, poor decision-making, and a lack of direction control (36), which increases the likelihood of involvement with violent scenarios, suicidal thoughts, and traffic accidents (33). This combination represents a serious public health problem because it potentiates clinical, mental, and social complications (37, 38). In addition, alcohol is often consumed to reduce anxiety and discomfort resulting from cocaine withdrawal (39, 40), and drug consumption favors contact with illegality and violence, thus maintaining the vicious circle.

Drug-using sexual partners was also independently associated with risky alcohol consumption. Drug users could encourage drug consumption in their partners (5, 40). According to Mburu et al. (41), partners who are drug users facilitate the initiation of substance use because their partners try to adopt a similar lifestyle and share something based on the ideal of social protection.

Most transgender women reported sex under the influence of alcohol, and they were 2.9-times more likely to participate in risky alcohol consumption. In Peru, Herrera et al. (29) reported a higher proportion of sex under the influence of alcohol among transgender women and the partners of transgender women compared to men who have sex with men. Alcohol causes modulating effects, altering sensations and decreasing body reflexes, as well as difficulty in making decisions, which can lead to risky sexual relationships, such as unprotected sex (42), and consequently a higher risk of HIV infection. A meta-analysis involving 10 studies showed that individuals who consumed alcohol prior to or during sex had an 87% increased risk of HIV infection. Moreover, among those who reported binge drinking, the risk was doubled in those who did not binge drink (43). Therefore, risky alcohol consumption may hinder efforts to control the HIV epidemic because of its negative effects on decisions regarding safe sex. Further, in the era of HIV pre-exposure prophylaxis, studies are needed to evaluate the adherence and effectiveness of this pharmacological preventive measure in transgender women under the influence of alcohol.

Furthermore, in the era of HIV pre-exposure prophylaxis.

### 4.1 Limitations

This study had some limitations. The cross-sectional design does not allow for the inference of causality, and the reverse effect of the risky behaviors on the alcohol risky consumption should not be discarded. The investigation was conducted in the state of Goiás, in the mid-western region of Brazil; therefore, the results may not be generalizable to all transgender women in Brazil or elsewhere. However, transgender women have social and demographic characteristics similar to those reported in other studies (2, 44–46).

Although it is unquestionable that RDS allows for the recruitment of individuals who would otherwise not be identified or reached via traditional sampling methods, the results cannot be generalized. It is possible that heavy drinking is a social network phenomenon; therefore, RDS would result in a sample restricted to this social network. Last, the interviews’ face-to-face behavioral variables could experience response bias owing to social desirability. We explored these potential biases and tried to minimize the effects through the instruments used in researchers’ data collection and the training of the interviewers.

## 5 Conclusion

Our findings suggest that transgender women are more likely to engage in high-risk alcohol use and are more exposed to factors that could promote the use of other hazardous substances than members of the general population, and that high-risk alcohol uses creates a vicious circle. Therefore, it should be a priority goal for health managers to develop strategies at the global level to monitor transgender women through services available in health networks to reduce their alcohol consumption and thereby improve the ability of health workers to care for this minority population.

## Data availability statement

The raw data supporting the conclusions of this article will be made available by the authors, without undue reservation.

## Ethics statement

This study was approved by the institutional research ethics committee (Protocol number. 2.358.818) of Universidade Federal de Goiás, Goiânia, Brazil. The studies were conducted in accordance with the local legislation and institutional requirements. Written informed consent for participation in this study was provided by the participants’ legal guardians/next of kin.

## Author contributions

LSM: Formal analysis, Conceptualization, Methodology, Writing–original draft (lead). KCS: Writing–review & editing (supporting). BVDS: Writing–review & editing (supporting); GFSF: Writing–review & editing (supporting). KV: Conceptualization, Writing–original draft (equal), Supervision. RICG: Writing–review & editing (supporting). SCP: Data curation, Conceptualization (supporting). MASC: Conceptualization, Investigation (equal). KAAC: Conceptualization, Investigation (equal). RLC: Formal analysis, Conceptualization, Methodology, Writing–original draft (lead), Supervision. SAT: Data curation, Investigation, Formal analysis, Funding acquisition, Conceptualization, Methodology, Writing–original draft (lead), Supervision.
